# Conformation of the von Willebrand factor/factor VIII complex in quasi-static flow

**DOI:** 10.1016/j.jbc.2021.100420

**Published:** 2021-02-16

**Authors:** Ernest T. Parker, Pete Lollar

**Affiliations:** Aflac Cancer and Blood Disorders Center, Children's Healthcare of Atlanta; Department of Pediatrics, Emory University, Atlanta, Georgia, USA

**Keywords:** von Willebrand factor, factor VIII, structural model, protein conformation, analytical ultracentrifugation, dynamic light scattering, size-exclusion chromatography, DLS, dynamic light scattering, EM, electron microscopy, MHKS, Mark–Houwink–Kuhn–Sakurada, PDI, polydispersity index, SEC, size-exclusion chromatography, SV, sedimentation velocity, SV AUC, sedimentation velocity analytical ultracentrifugation, VWF, Von Willebrand factor

## Abstract

Von Willebrand factor (VWF) is a plasma glycoprotein that circulates noncovalently bound to blood coagulation factor VIII (fVIII). VWF is a population of multimers composed of a variable number of ∼280 kDa monomers that is activated in shear flow to bind collagen and platelet glycoprotein Ibα. Electron microscopy, atomic force microscopy, small-angle neutron scattering, and theoretical studies have produced a model in which the conformation of VWF under static conditions is a compact, globular “ball-of-yarn,” implying strong, attractive forces between monomers. We performed sedimentation velocity (SV) analytical ultracentrifugation measurements on unfractionated VWF/fVIII complexes. There was a 20% per mg/ml decrease in the weight-average sedimentation coefficient, *s*_*w*_, in contrast to the ∼1% per mg/ml decrease observed for compact globular proteins. SV and dynamic light scattering measurements were performed on VWF/fVIII complexes fractionated by size-exclusion chromatography to obtain *s*_*w*_ values and z-average diffusion coefficients, *D*_*z*_. Molecular weights estimated using these values in the Svedberg equation ranged from 1.7 to 4.1 MDa. Frictional ratios calculated from *D*_*z*_ and molecular weights ranged from 2.9 to 3.4, in contrast to values of 1.1–1.3 observed for globular proteins. The Mark–Houwink–Kuhn–Sakurada scaling relationships between *s*_*w*_, *D*_*z*_ and molecular weight, $s=k^{\prime }M^{a_{s}}$ and $D=k^{\prime \prime }M^{a_{D}}$, yielded estimates of 0.51 and –0.49 for *a*_*s*_ and *a*_*D*_, respectively, consistent with a random coil, in contrast to the *a*_*s*_ value of 0.65 observed for globular proteins. These results indicate that interactions between monomers are weak or nonexistent and that activation of VWF is intramonomeric.

Von Willebrand factor (VWF) is a large plasma glycoprotein that participates in platelet adhesion and aggregation and serves as a transport protein for blood coagulation factor VIII (fVIII). The participation of VWF in platelet function and fVIII transport is necessary for normal hemostasis. Decreased levels or dysfunctional VWF collectively produce the disorder von Willebrand disease. In its mild form, von Willebrand disease is the most common congenital bleeding disorder (1). Severe von Willebrand disease is a life-threatening disorder that, in addition to a marked inability to support platelet function, produces a hemophilia A-like syndrome due to low levels of fVIII.

VWF is synthesized in endothelial cells, where it is either secreted constitutively or stored in Weibel–Palade bodies, and in megakaryocytes, where it is stored in α-granules (2). VWF is a multimer consisting of a linear chain of covalently linked ∼277 kDa subunits called monomers. It is intrinsically heterogeneous with respect to the number of monomers per multimer (2, 3, 4). A mean of eight monomers has been estimated by rotary shadowed electron microscopy (EM), corresponding to a molecular weight of ∼2.2 MDa (5). However, plasma-derived multimers as large as 10 MDa have been observed (6). VWF in Weibel–Palade bodies is even larger than plasma VWF and contains 60–250 monomers per “ultralarge” multimer with lengths up to 15 μm (4).

The VWF monomer propeptide contains a sequence of domains designated D1–D2–D′–D3–A1–A2–A3–D4–B1–B2–B3–C1–C2–CK (3). The propeptide is cleaved between the D2 and D′ domains to produce the mature VWF monomer. The monomers are disulfide-linked at their C-terminal ends to form dimers, which in turn are disulfide-linked at their N-terminal ends to form multimers (3). VWF contains 19% carbohydrate by mass (7), which includes extensive O-glycosylation, primarily at interdomain segments at the N- and C-terminal ends of the A1 domain (8). X-ray structures are available for the D′D3, A1, A2, and A3 domains and reveal that they are globular, ∼30–40 kDa proteins (9, 10, 11, 12).

Following vascular injury in shear flow, VWF binds to exposed subendothelial collagen and mediates platelet adhesion by binding platelet glycoprotein Ibα (1). It also participates in platelet aggregation by binding to the platelet integrin receptor αIIbβ3. VWF multimers elongate under shear forces in flowing blood. In cultured endothelial cell monolayers in shear flow, VWF forms “strings” exceeding 1 mm in length, which have been proposed to be either ultralarge multimers or the product of multimer self-association (13). Ultralarge multimers are proteolytically cleaved by ADAMTS-13 to yield the distribution of multimers that is observed in plasma and in purified preparations of VWF, including VWF/FVIII products used to treat von Willebrand disease and hemophilia A. Congenital or autoimmune-mediated deficiency of ADAMTS-13 results in the appearance of ultralarge VWF multimers and produces the disorder thrombocytopenic thrombotic purpura (14, 15).

Based on EM, atomic force microscopy, small-angle neutron scattering, total internal reflection fluorescence microscopy, and theoretical studies, the conformation of VWF under static conditions has been variably described as a “ball-of-yarn” (16), a “tangled coil” (6), a “compact fuzz ball” (17), a “compact, bird's nest” (4), “compact and globular” (18), and a “dense globule” (19). These descriptions suggest that the conformation of VWF/fVIII is a function of strong attractive forces between monomers. The unfolding of the compact state in shear flow is considered an essential feature of the activation of VWF to support platelet function.

An alternative conformation is a random coil, or synonymously, a flexible chain, defined as a chain in which one of its elements can have any direction relative to another element if the distance between elements is large enough (20). The distinction between globular and flexible conformations can be resolved using hydrodynamic methods. These methods are particularly useful if a homologous series of polymers is available for study and have been applied extensively to the study of the conformation of synthetic polymers, polysaccharides, and nucleic acids (20, 21, 22, 23). In this paper, the hydrodynamic properties of unfractionated VWF/FVIII and on a homologous series of fractionated VWF/fVIII multimers were characterized by sedimentation velocity analytical ultracentrifugation (SV AUC) in quasi-static flow and by dynamic light scattering (DLS). In contrast to a compact, globular conformation, our results are consistent with a random coil conformation and the absence of intermonomeric interactions.

## Results

### SV AUC of unfractionated VWF/fVIII complexes

**Figure 1:**
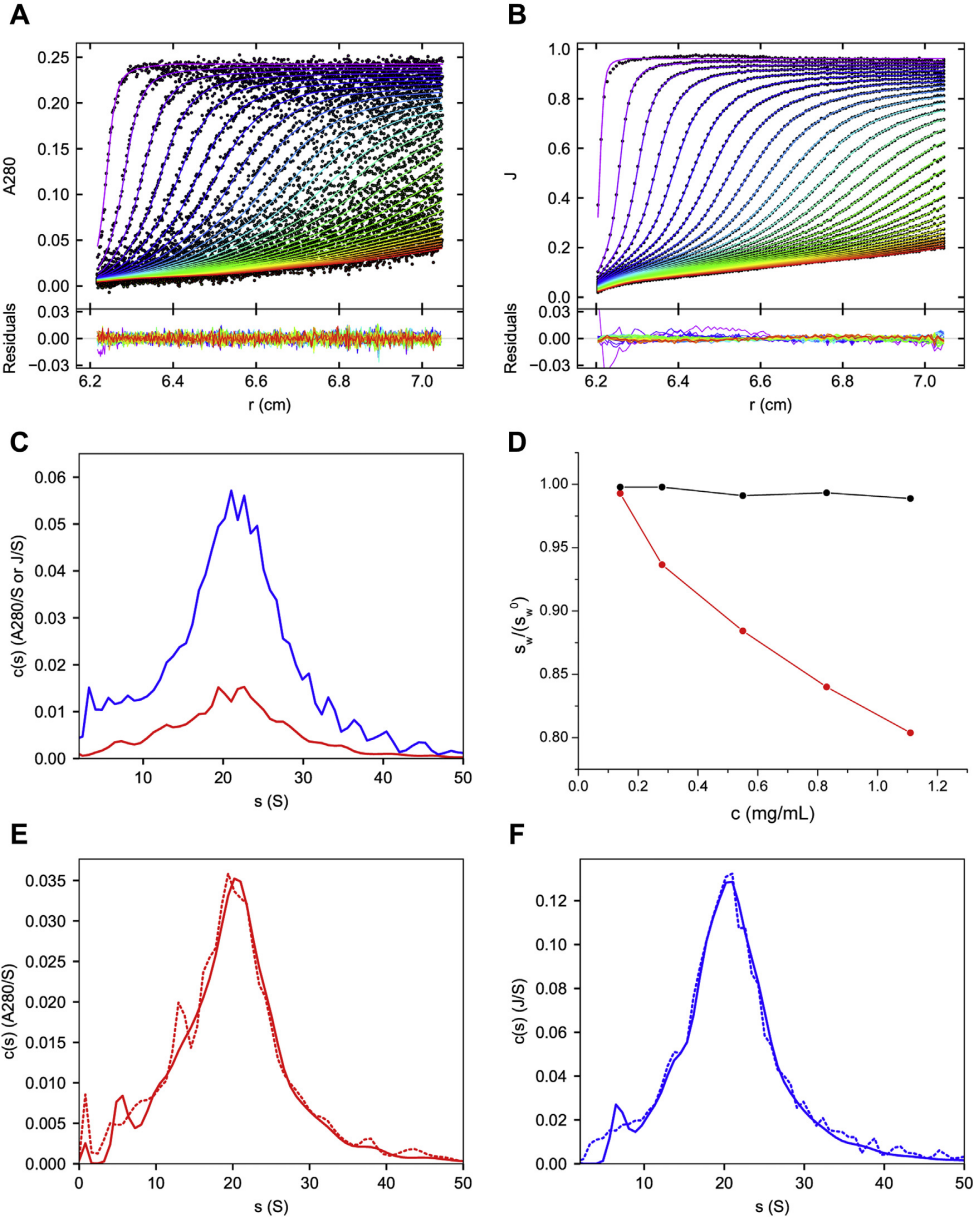
**SV AUC of unfractionated VWF/fVIII complexes**. *A* and *B*, Heparin-Sepharose purified VWF/FVIII, 0.28 mg/ml in HBS/Ca Buffer was centrifuged at 45,400*g* in a Beckman-Coulter XLI analytical ultracentrifuge as described in Experimental procedures. The data traces from left to right are scans taken at increasing times during centrifugation. *A*, absorbance scans at 280 nm. *B*, interference scans; J, fringe increment. Only every tenth data point of the interference scans is shown for clarity. Curves represent fits to the continuous c(s) distribution model in SEDFIT. The root-mean-square deviations of the fits are 0.0040 and 0.0029 for A280 and interference data, respectively. *C*, c(s) distributions derived from the fitted data. *Red*, A280; *blue*, interference. *D*, unfractionated VWF/FVIII (*red*) and bovine serum albumin (*black*) were centrifuged at 45,400*g* and 182,000*g*, respectively, at concentrations ranging from 0.14 to 1.1 mg/ml. *s*_*w*_ values were calculated from c(s) distributions derived from A280 data. *s*_*w*_ values for bovine serum albumin were obtained by integrating the monomer peak of the c(s) distribution (75). The value at infinite dilution, $s_{w}^{0}$, was obtained by regression analysis. *E–F*, VWF/FVIII, 0.55 mg/ml in HBS/Ca Buffer was centrifuged at either 16,400*g* (15,000 RPM), dotted lines or 45,400*g* (25,000 RPM), solid lines. *E*, A280 data, *F*, interference data.

VWF/fVIII complex was purified from the commercial, plasma-derived product, Alphanate, by heparin-Sepharose chromatography as described in Experimental procedures and Supporting information (Fig. S1). The resulting preparation, containing unfractionated VWF/fVIII complexes, was subjected to SV AUC at 45,400*g*. Absorbance at 280 nm (Fig. 1*A*) and the interference fringe increment (Fig. 1*B*) were measured as a function of time and radial position. The data were analyzed using the continuous c(s) distribution model in SEDFIT as described in Experimental procedures and Supporting information. Excellent fits were obtained on the order of the random noise in the data acquisition. Although only a single boundary is evident in Figure 1, *A* and *B*, the broad c(s) distributions revealed a polydisperse population of species sedimenting predominantly between 10 and 30 S (Fig. 1*C*).

Diffusion contributes to the shape of the sedimenting boundary for particles that diffuse on a timescale of sedimentation. The continuous c(s) distribution model attempts to deconvolute the contribution of diffusion to the boundary structure. It utilizes the functional relationship between the diffusion coefficient and the frictional ratio for a single species and assumes that a common frictional ratio can be used as a fitted parameter as described in Supporting information (24, 25, 26). For monodisperse systems, the model provides accurate measurements of the sedimentation coefficient, frictional ratio, and diffusion coefficient, as judged by its ability to estimate molecular weights accurately using the Svedberg equation (25).

In polydisperse systems, the model captures some elements of the diffusion process and returns a single weighted frictional ratio, but molecular weights generally cannot be estimated accurately. Nonetheless, the weight-average sedimentation coefficient, *s*_*w*_, of a polydisperse system can be measured using the continuous c(s) distribution model (27, 28). For polydisperse systems consisting of species with variable extinction or fringe coefficients, *s*_*w*_ is a signal-average value and not a true weight-average. However, for VWF/fVIII, the extinction coefficient at 280 nm and the fringe extinction coefficient are constant for all multimers and equal to that of the monomer, allowing measurement of true *s*_*w*_ values. Integration of the c(s) distributions in Figure 1*C* and adjusting the resulting *s*_*w*_ values to the standard condition, ${\left(s_{w}\right)}_{20,w}$, of 20 °C in solvent water yielded values of 23.2 S and 23.5 S by absorbance and interference measurements, respectively.

The data in Figure 1, *A* and *B* were also fitted to the ls-g∗(s) model in SEDFIT, which assumes no diffusion during the centrifugation process (24). Although *s*_*w*_ values were produced that were close to the values in the continuous c(s) distribution model, the fits were poorer at the 95% confidence level as judged by F-statistics of the rmsd ratios (29) using the variance ratio calculator in SEDFIT. Thus, the continuous c(s) distribution model was used for the remainder of the study.

In the absence of self-association, the sedimentation coefficients of macromolecules decrease with increasing concentration, largely due to the effects of solution nonideality on the frictional coefficient (21). ${\left(s_{w}\right)}_{20,w}$ values of unfractionated VWF/fVIII were measured as a function of loading concentrations from 0.14 to 1.1 mg/ml and decreased by ∼20% per mg/ml (Fig. 1*D*). In contrast, the sedimentation coefficient of bovine serum albumin decreased by only ∼1% per mg/ml (Fig. 1*D*) in agreement with the decrease typically observed for compact, globular proteins (30), which is close to the theoretical value of 0.5% per mg/ml for a sphere with a density of 1.4 mg/ml (31). Thus, the concentration-dependent decrease in *s*_*w*_ for unfractionated VWF/fVIII is inconsistent with a compact, globular conformation. Additionally, there was no evidence for self-association, which manifests as an increase in sedimentation coefficient with concentration.

Sedimentation in the ultracentrifuge produces quasi-static flow in which centrifugal motion of the macromolecule is matched by centripetal flow of displaced solvent. The scans shown in Figure 1, *A* and *B* taken over 4 h of centrifugation show that the velocity of VWF/fVIII as revealed by boundary migration is less than 1 cm per hour. The frictional force experienced by a particle during centrifugation is the product of the frictional coefficient and velocity, *fv*, and because of the low velocity is typically only on the order of fN (32). However, long linear macromolecules can undergo a conformational change due to the frictional forces experienced during ultracentrifugation, which manifests as a decrease in the sedimentation coefficient with increasing rotor speed (33). Thus, the *s*_*w*_ of unfractionated VWF/FVIII complexes was investigated by comparing c(s) distributions at 16,400*g* (15,000 RPM) and 45,400*g* (25,000 RPM). Figure 1, *E* and *F* show that the c(s) distributions are superimposable at the two rotor speeds. This result indicates that the conformation of VWF/FVIII is not altered by ultracentrifugation and is consistent with the fact that conformational changes during ultracentrifugation have only been observed for particles with molecular weights greater than 100 MDa (33).

### SEC fractionation of VWF/fVIII complexes

**Figure 2:**
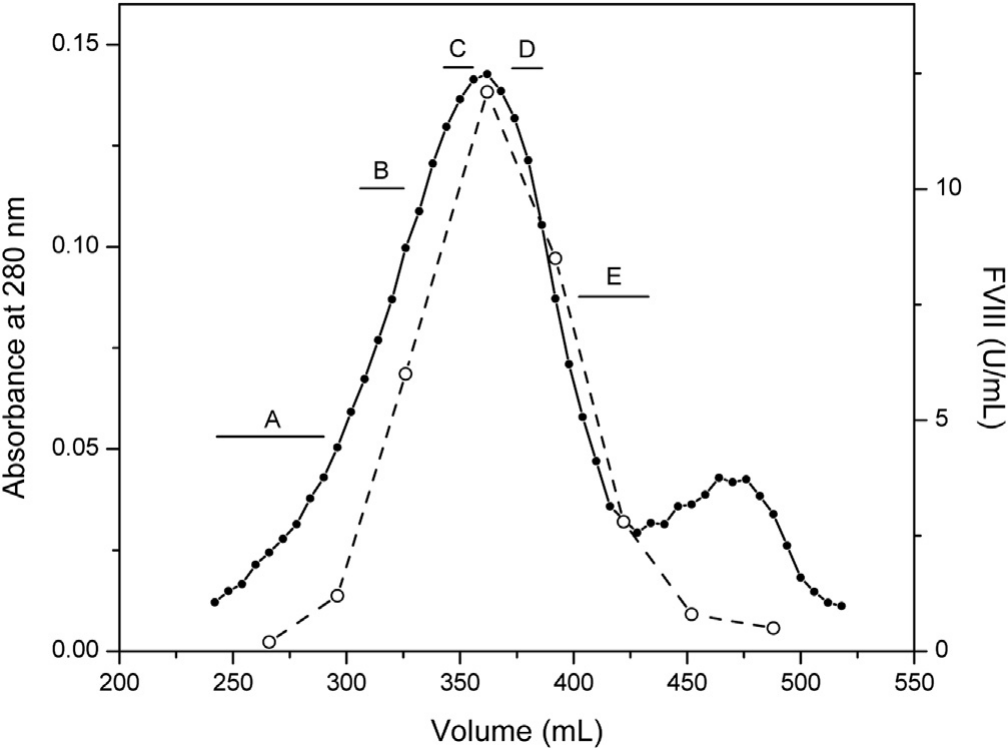
**SEC of VWF/fVIII complexes**. Heparin-Sepharose purified VWF/fVIII underwent Sephacryl S-1000 SEC as described in Experimental procedures. Closed circles, A280; open circles, fVIII activity. Fractions corresponding to the elution volumes shown in the figure, designated Samples A, B, C, D, and E, respectively, were pooled, exchanged into HBS/Ca Buffer, and concentrated by repeated ultrafiltration.

VWF/fVIII multimers are an example of a homologous series of polymers, analogous to synthetic polymers, polysaccharides, and nucleic acids. The hydrodynamic characterization of partially fractionated homologs is a powerful method to assess solution conformation (20, 21, 22, 23). VWF/fVIII was fractionated by Sephacryl S-1000 size-exclusion chromatography (SEC) as described in Experimental procedures. Figure 2 shows the resulting chromatogram, revealing a broad distribution due to the polydispersity of VWF multimers. SEC fractions were pooled to produce samples labeled A through E as indicated.

FVIII activity was present across the distribution up to a trailing minor peak containing material that has not been identified. The concentrations of VWF and fVIII activity in the heparin-Sepharose purified starting material were 0.54 mg/ml and 38 U/ml, respectively, yielding a specific fVIII activity of 70 U/mg. Since the specific activity of fVIII in the absence of VWF is ∼5000 U/mg (34), the percentage mass ratio of fVIII to VWF is ∼1%. This approximates the probability that a vWf monomer contains a bound fVIII since the molecular weights of the VWF monomer and fVIII are nearly equal. The median molecular weight of a plasma-derived VWF multimer has been estimated to be 2.2 MDa by EM. Since Figure 2 indicates that fVIII is randomly distributed across the multimer population, a 2.2 MDa 8-mer has only a ∼10% chance of being occupied by fVIII. Thus, the hydrodynamic properties of the multimer population are unlikely to be influenced by fVIII.

### Sedimentation coefficients of SEC-fractionated VWF/fVIII complexes

**Figure 3:**
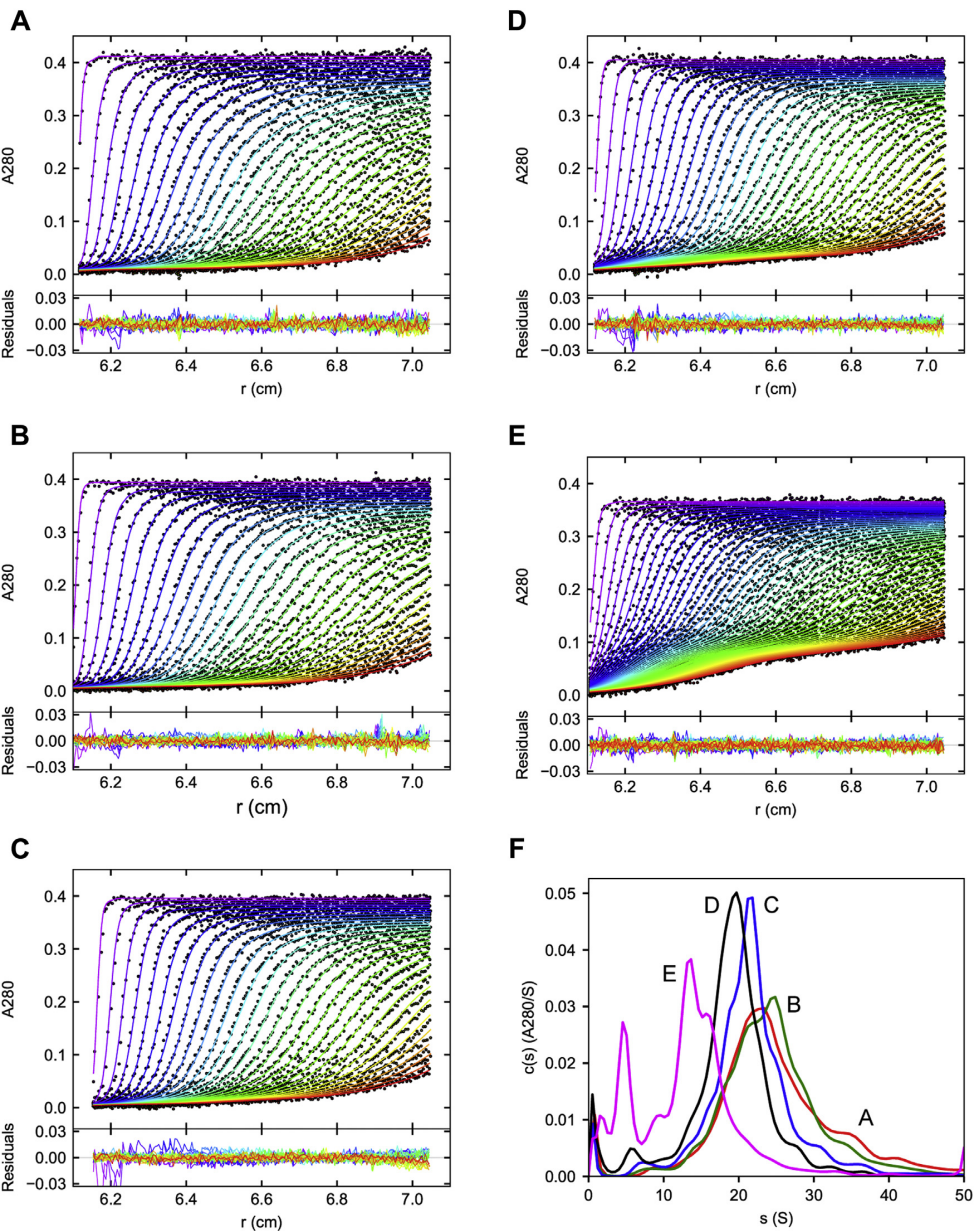
**SV AUC of SEC-fractionated VWF/fVIII complexes**. SEC Samples A–E in HBS/Ca Buffer were centrifuged at 45,400*g*. *A–E*, absorbance scans at 280 nm. Curves represent fits to the continuous c(s) distribution model in SEDFIT. Only every other data point is shown for clarity. The root-mean-square deviations of the fits ranged from 0.0040 to 0.0045. *F*, c(s) distributions derived from the fitted data.

SEC-fractionated VWF/fVIII complexes were subjected to SV AUC as described in Experimental procedures. Figure 3, *A*–*E* shows fits of absorbance data to the continuous c(s) distribution model. The resulting c(s) distributions are shown in Figure 3*F*, which were integrated to produce ${\left(s_{w}\right)}_{20,w}$ values. As expected, ${\left(s_{w}\right)}_{20,w}$ decreased with increasing elution volume, ranging from 27.7 to 16.8 S (Table 1). In contrast to Samples A–D, in which only a single boundary was discernable, the scans for Sample E revealed at least two boundaries and c(s) distribution analysis identified a minor ∼5 S peak (Fig. 3, *E* and *F*). The width of the c(s) distributions in Figure 3*F* indicates that there is significant polydispersity since the peak width of monodisperse samples typically is less than 1 S at similar loading concentrations. Interference scans were obtained during the same run and yielded ${\left(s_{w}\right)}_{20,w}$ values within 1.0 S of the absorbance data for all samples (Supporting information, Fig. S2 and Table 1). Although still polydisperse, these results indicate that SEC fractionation provides resolution of the sedimentation behavior of VWF/fVIII complexes.

**Table 1:** SV and DLS analysis of SEC-fractionated VWF/fVIII complexes

Sample	(s_w_)_20,w_ (S)		(D_z_)_20,w_ × 10^7^ (cm^2^ s^−1^)	PDI	Rh (nm)	MW (MDa)		f/f_o_	
	A280	IF				A280	IF	A280	IF
A	27.74 (27.52, 27.97)	26.81 (27.74, 26.88)	0.620 (0.609, 0.631)	0.26	32.3	4.10	3.92	3.37	3.42
B	25.80 (25.63, 26.96)	25.65 (25.59, 25.75)	0.650 (0.647, 0.653)	0.24	30.8	3.66	3.59	3.33	3.36
C	23.27 (23.11, 23.44)	23.24 (23.19, 23.32)	0.712 (0.706, 0.718)	0.24	28.2	3.04	2.99	3.22	3.26
D	20.45 (20.37, 20.62)	20.70 (20.65, 20.75)	0.792 (0.787, 0.797)	0.16	25.3	2.43	2.42	3.10	3.15
E	16.84 (16.73, 16.99)	16.80 (16.70, 16.95)	0.946 (0.943, 0.949)	0.24	21.2	1.71	1.68	2.90	2.98

A280, absorbance at 280 nm; IF, interference.

Numbers in parentheses represent 95% confidence intervals.

(s_w_)_20,w_: Weight-average sedimentation coefficient adjusted the standard condition of 20 °C in water.

(D_z_)_20,w_: z-average diffusion coefficient adjusted to the standard condition of 20 °C in water.

PDI: polydispersity index (Equation 6).

Rh: Hydrodynamic radius (Equation 7).

f/f_o_: frictional ratio (Equations 8 and 9).

### Diffusion coefficients and hydrodynamic radii of SEC-fractionated VWF/fVIII complexes

**Figure 4:**
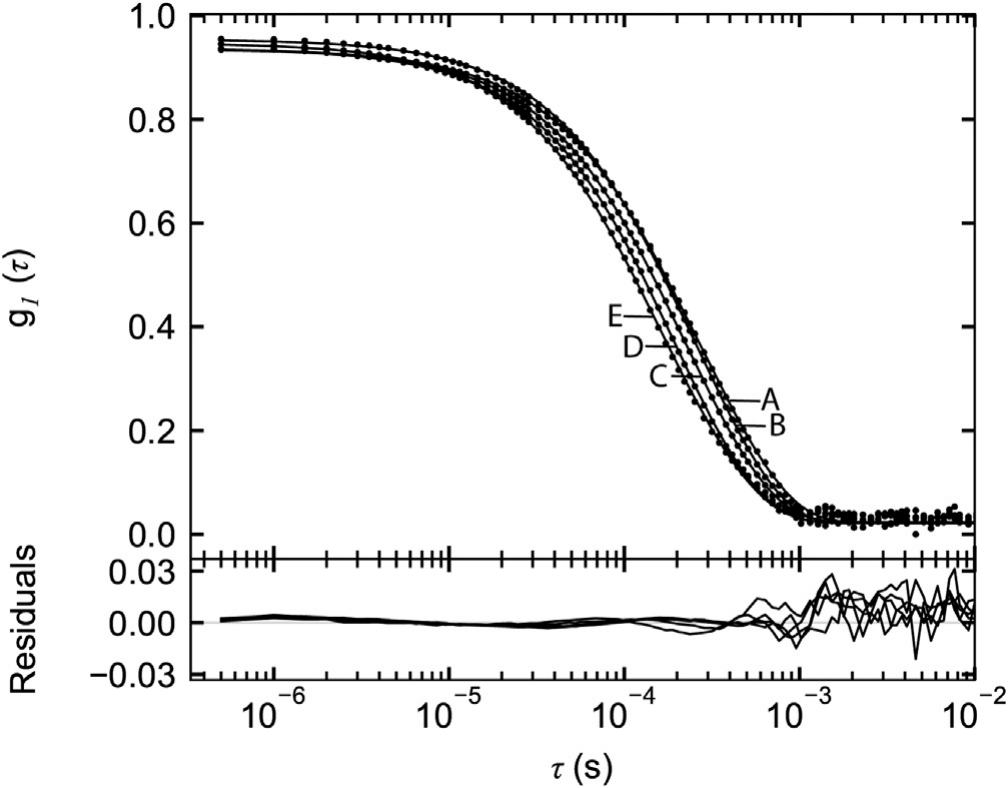
**DLS of SEC fractionated VWF/fVIII complexes**. DLS measurements were made on Sephacryl S-1000 SEC Samples A–E in HBS/Ca Buffer as described in Experimental procedures. The normalized electric field correlation function, $g_{1}\left(\tau \right)$, *versus* decay time, *τ*, is shown for the median decay rate of eight measurements for each sample. The curves represent fits to the cumulants analysis model in SEDFIT. The first and second moments of the cumulants fit were used to calculate the z-average diffusion coefficients and polydispersity indices in Table 1.

DLS measurements were made on SEC Samples A–E to obtain estimates of the z-average diffusion coefficients and hydrodynamic radii. Figure 4 shows the fits of the normalized electric field correlation function, $g_{1}\left(\tau \right)$, to decay times using cumulants analysis. The decay curves shift from right to left from A to E corresponding to increasing SEC elution volume, consistent with faster diffusion and smaller hydrodynamic radii of the eluting species. The z-average diffusion coefficients, ${\left(D_{z}\right)}_{20,w}$, obtained from the first moment of the cumulants analysis, ranged from 0.620 × 10^−7^ to 0.946 10^−7^ cm^2^ s^−1^ (Table 1). The polydispersity indices (PDI) ranged from 0.16 to 0.26 (Table 1). Values below 0.15 are considered consistent with monodispersity (35). Thus, the polydispersity reflects incomplete separation of the VWF multimers, consistent with the SV results in Figure 3. Hydrodynamic radii were calculated using the Stokes–Einstein equation (Equation 7) and ranged from 21.2 to 32.3 nm (Table 1).

### Molecular weights and frictional ratios of SEC-fractionated VWF/fVIII complexes

SEC fractionation of VWF/fVIII complexes provided sufficient resolution to produce distinct *s*_*w*_ and *D*_*z*_ values (Table 1). Estimates of sedimentation and diffusion coefficients at infinite dilution, ${\left(s_{w}^{0}\right)}_{20,w}$ and ${\left(D_{z}^{0}\right)}_{20,w}$, were made as described in Experimental procedures and used to calculate molecular weights using the Svedberg equation (Equation 14) with the caveat that combining weight- and z-averages produces a molecular weight estimate that is neither a weight- or z-average. Molecular weight estimates ranged from 1.7 to 4.1 MDa (Table 1). Using a molecular weight of 277 kDa for the mature VWF monomer based on amino acid sequence and carbohydrate composition (7, 8), the average number of VWF monomers in Samples E and A is 6 and 15, respectively.

Frictional coefficients for Sephacryl S-1000 SEC Samples A–E were calculated from the experimental diffusion coefficients (Table 1) using the Einstein diffusion equation (Equation 8). The frictional coefficient of a sphere having the same anhydrous molecular weight and specific volume of the particle, *f*_*o*_, was calculated using the molecular weights in Table 1 and the partial specific volume (Equation 9). The frictional ratio, *f*/*f*_*o*_, is a measure of departure from spherical geometry and/or hydration of the protein (36). Frictional ratios for globular proteins range from 1.1 to 1.3 (Supporting information, Table S1). The values for SEC Samples A–E, ranging from 2.9 to 3.4, were considerably larger (Table 1), indicating that VWF/fVIII complexes are not compact, globular structures.

**Figure 5:**
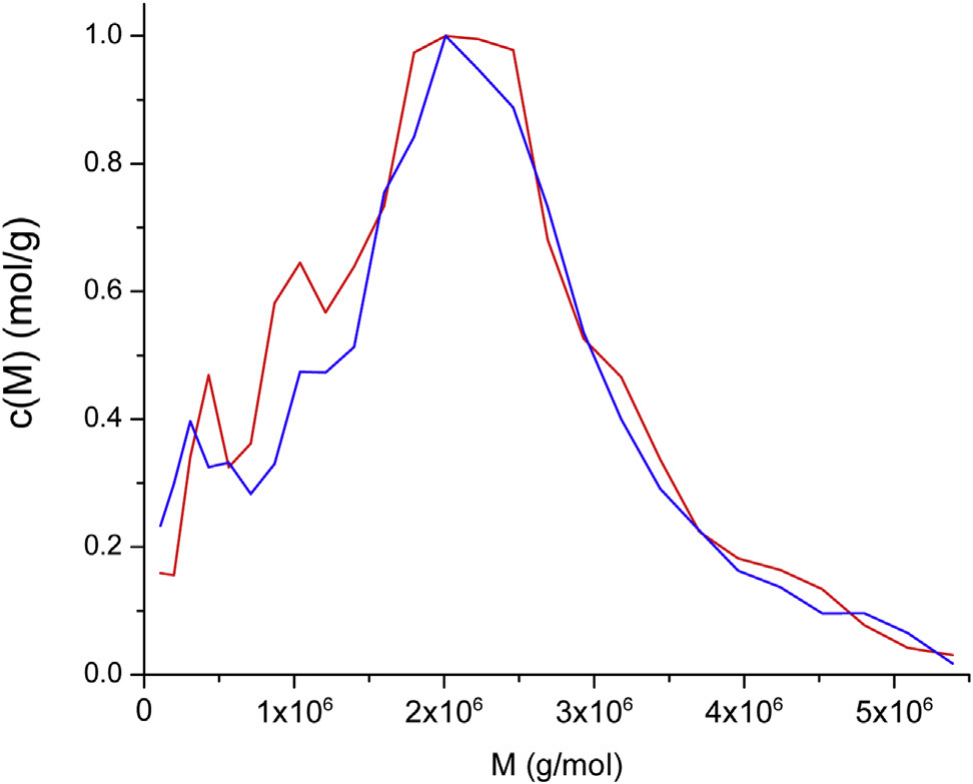
**c(M) distribution of unfractionated VWF/fVIII complexes**. The A280 scans (Fig. 1*A*) and interference scans (Fig. 1*B*) of heparin-Sepharose purified VWF/FVIII were fit the continuous c(M) distribution model in SEDFIT using a fixed frictional ratio of 3.0 as described in Experimental procedures. *Red*, absorbance; *blue*, interference. c(M) values are normalized to the maximum value.

Although the molar mass distribution of a polydisperse population cannot be estimated using an *a priori* assumption of single common frictional ratio, the frictional ratios estimated with the aid of the DLS measurements ranged from only 2.9 to 3.4 (Table 1). This allowed an estimation of the molar mass distribution by fixing the frictional ratio at 3.0 and fitting A280 scans of unfractionated VWF/fVIII (Fig. 1, *A* and *B*) to the continuous c(M) distribution model in SEDFIT (24) as described in Experimental procedures. This analysis revealed a unimodal distribution with a median molecular weight of ∼2.2 MDa (Fig. 5).

### Relationships between sedimentation and diffusion coefficients and molecular weight of fractionated VWF/fVIII complexes

**Figure 6:**
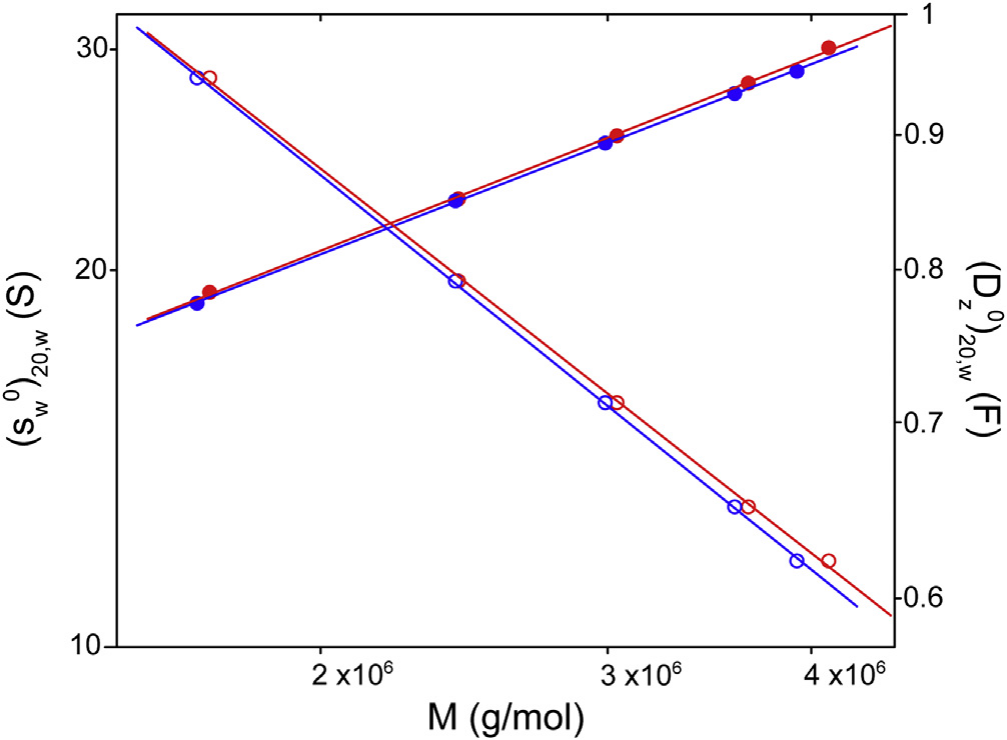
**Sedimentation coefficient and diffusion coefficient scaling factors for fractionated VWF/fVIII complexes**. ${\left(s_{w}^{0}\right)}_{20,w}$ (*closed circles*) and ${\left(D_{z}^{0}\right)}_{20,w}$ (*open circles*) *versus M* values for A280 data (*red symbols*) and interference data (*blue symbols*) (Table 1) are plotted on logarithmic scales along with linear regression lines. The units of *D*_*z*_ are Ficks (1 F = 10^–7^ cm^2^/s). The slopes and 95% confidence limits for the A280 *s*_*w*_ and *D*_*z*_ regressions are 0.51 (0.49,0.53) and –0.49 (–0.47,–0.51), respectively. The corresponding values for the interference regressions are 0.50 (0.48,0.52) and –0.50 (–0.48,–0.52), respectively.

Sedimentation coefficients and diffusion coefficients are related to molecular weight by the Mark–Houwink–Kuhn–Sakurada (MHKS) relationships, $s=k^{\prime }M^{a_{s}}$and $D=k^{\prime \prime }M^{a_{D}}$, where the scaling factors, *a*_*s*_, and *a*_*D*_, depend on macromolecular conformation (20, 21, 22, 23). The scaling factors for spheres, random coils, and rods are given in Table 2. Figure 6 shows plots of log *s*_*w*_ and log *D*_*z*_ *versus* log *M* for fractionated VWF/fVIII complexes obtained from absorbance and interference data (Table 1). The values of *a*_*s*_ and *a*_*D*_ estimated from the slopes of the linear regression lines for the A280 data are 0.51 and –0.49, respectively. Values obtained from interference data were 0.50 and –0.50. These results are consistent with a random coil conformation for VWF/fVIII complexes within a range of molecular weights from 1.7 to 4.1 MDa.

**Table 2:** Hydrodynamic scaling laws

Shape	*a*_*s*_	*a*_*D*_
Sphere	0.67	−0.33
Coil	0.4–0.5	−(0.5–0.6)
Rod	0.2	−0.85

From (22, 23).

$s=k^{\prime }M^{a_{s}}$.

$D=k^{\prime \prime }M^{a_{D}}$.

**Figure 7:**
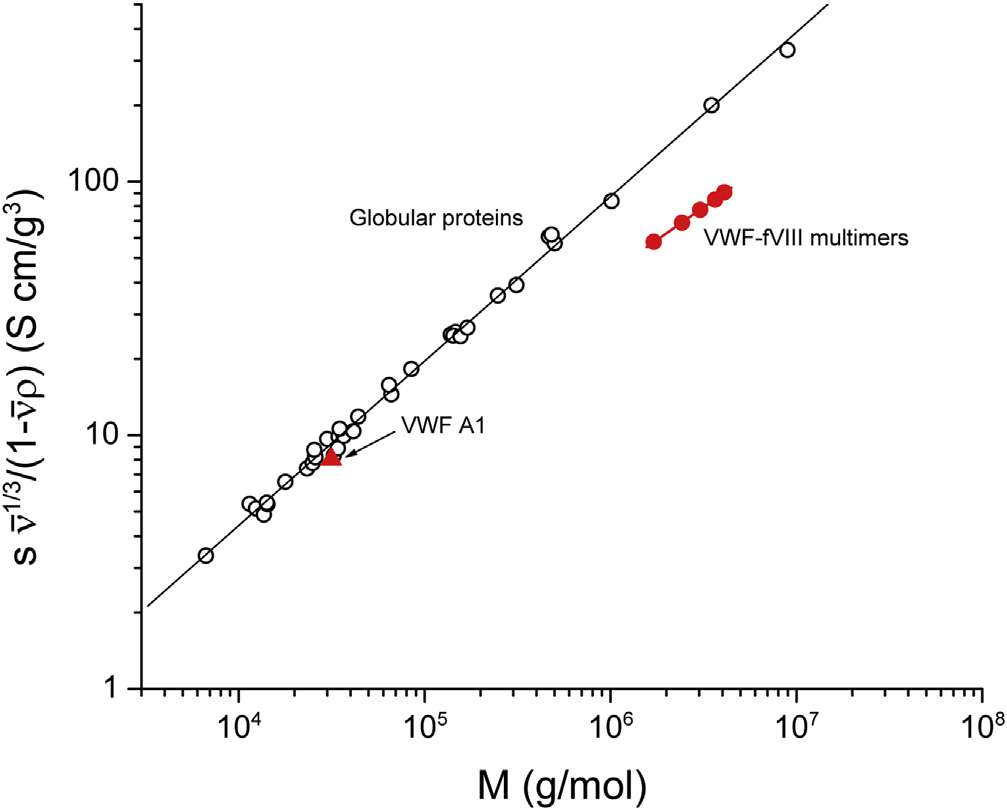
**Scaling factors for globular proteins compared to VWF/fVIII complexes.** Sedimentation coefficients, molecular weights, and partial specific volumes for a series of globular proteins (*open black circles*) are listed in Table S1. The slope of the regression line for globular proteins is 0.65. The data for VWF/fVIII (*closed red circles*) are derived from A280 SV AUC data (Table 1). Data for the VWF A1 domain (*red triangle*) is from (63).

The sedimentation coefficient relationship for VWF/fVIII complexes is compared with the relationship for globular proteins in Figure 7. The data are normalized with respect to partial specific volume (37), which varies among the globular proteins. The slope of the regression line for globular proteins is 0.65, consistent with nearly spherical geometry, and significantly different from the slope for VWF/fVIII complexes. The figure also illustrates the relatively small sedimentation coefficients of VWF/fVIII complexes compared with globular proteins of equal molecular weight. For example, a globular protein with the same molecular weight and partial specific volume as 23 S VWF/fVIII would sediment at 60 S. Also shown in Figure 7 is the relationship between the sedimentation coefficient and molecular weight for the ∼30 kDa vWf A1 domain, which behaves hydrodynamically as a globular protein.

Examples of MHKS sedimentation velocity exponents, *a*_*s*_, obtained from the literature are shown in Table 3 for comparison with the current study. Glycogen has a globular, compact structure. Collagen and schizophyllum are well-known examples of rod-like macromolecules. The *a*_*s*_ of DNA increases with molecular weight as it progresses from a relatively stiff chain to a random coil. Heparin is a semiflexible with an *a*_*s*_ value of 0.38. Amylose has a random coil conformation. Globular proteins unfold in guanidine hydrochloride under reducing conditions into a random coil conformation in which the coil is the polypeptide chain. In contrast, the VWF/fVIII coil presumably is a chain of globular domains and possibly disordered interdomain linking sequences that make up the VWF monomer. Mucins are another possible example of coils made up of a string of domains.

**Table 3:** MHKS sedimentation velocity exponents

Molecule	Solvent	*a*_*s*_	Reference
Collagen	Aqueous	0.18	(60), Table I
Schizophyllum	Aqueous	0.23	(81), Tables I,II
DNA (3.3 kDa–1.6 MDa)	Aqueous	0.30	(48), Table S-3
Heparin	Aqueous	0.38	(82), Table 1
Polyisobutylene	Cyclohexane	0.40	(20), Table 22–2
DNA (3.2–120 MDa)	Aqueous	0.41	(48), Table S-3
Porcine submaxillary mucin	Aqueous	0.42	(50), Figure 1^a^
Globular proteins	GuCl/β-ME^b^	0.48	(83), Table II
Amylose	DMSO^c^	0.48	(84), Tables I,II
VWF/fVIII	Aqueous	0.51	This study
Globular proteins	Aqueous	0.65	(85), Table D2.3
Glycogen	Aqueous	0.70	(86), Table 1

a Combined sedimentation and diffusion data using $a_{s}=1+a_{D}$ (23).

b Guanidine hydrochloride, β-mercaptoethanol.

c Dimethylsulfoxide.

## Discussion

The compact globule, random coil, and rod represent the three archetypical macromolecular conformations (38). A globule-to-coil-to-rod spectrum exists that is governed by the interactions, or lack thereof, between the macromolecule and solvent and within the macromolecule itself. Globular, quasi-spherical proteins represent one extreme, in which attractive intramolecular forces and hydrophobic effects collapse the polypeptide chain into a folded, compact structure. It is followed by the flexible, random coil, which forms in the absence of intramolecular forces and in the presence of favorable interactions with solvent. At the other extreme, repulsive intramolecular forces produce a rod-like stiff chain.

Although the structure of the VWF/fVIII complex typically is characterized as a compact globule (4, 6, 16, 17, 18, 19, 39, 40, 41), our results strongly support a random coil conformation. We obtained the weight-average sedimentation coefficient, *s*_*w*_, of unfractionated VWF/fVIII complexes and *s*_*w*_ values and z-average diffusion coefficients, *D*_*z*_, of size-fractionated VWF/fVIII complexes. We observed a large, ∼20% per mg/ml decrease in *s*_*w*_ with increasing loading concentrations of unfractionated VWF/fVIII (Fig. 1*D*), which is over an order of magnitude greater than that observed for compact, globular proteins (30). The decrease in sedimentation coefficient with concentration is due to solution nonideality, largely due the effect of excluded volume on the frictional coefficient (21). Large *s*_*w*_ decreases are a characteristic of macromolecules exhibiting random coil behavior, including synthetic polymers (21), polysaccharides (42), and DNA (43).

The *s*_*w*_ and *D*_*z*_ values were used to estimate the molecular weights of fractionated VWF/fVIII complexes using the Svedberg equation (Equation 14). *D*_*z*_ values and molecular weights then were used to estimate the frictional ratios of fractionated VWF/fVIII using the Einstein diffusion equation (Equation 8) and the equation for the equivalent anhydrous sphere (Equation 9). The observed frictional ratios of ∼3 (Table 1) are considerably larger than the values of 1.1–1.3 that are observed for globular proteins (Supporting information, Table S1). These large frictional ratios are consistent with the concentration-dependent decrease in sedimentation coefficient, which scales approximately with the cube power of the frictional ratio (44, 45).

For a homologous series of polymers, the relationship between molecular weight and the classic hydrodynamic parameters—sedimentation coefficient, diffusion coefficient (or equivalently, the hydrodynamic radius), intrinsic viscosity, and radius of gyration—can be assessed usingMHKS scaling factors as a diagnostic for solution conformation (22, 23). The MHKS sedimentation coefficient and diffusion coefficient scaling factors for fractionated VWF/fVIII complexes of approximately 0.5 and –0.5 (Fig. 6) are consistent with the scaling factors for a random coil (Tables 2 and 3). These results can be compared with the sedimentation coefficient MHKS scaling factor for globular proteins of 0.65 (Fig. 7), which closely agrees with the 2/3 power law for a sphere (Table 2). The nonglobular nature of VWF/fVIII complexes is also evident from their relatively small sedimentation coefficients at equivalent molecular weights of the globular proteins (Fig. 7), which again is a manifestation of their large frictional ratios.

Random coils arise from rotation of segments of the coil in solution, producing a restricted random walk in space. All coils have some degree of stiffness because there is never completely free rotation of all segments. However, segments that are sufficiently far apart with respect to the stiffness of the chain have no memory of each other. Random coils have been modeled as worm-like semiflexible cylinders (23, 38, 46, 47) or strings of beads (48). For synthetic polymers, polysaccharides, and nucleic acids, the diameter of the cylinder is due to the size of the repeating units (*e.g.*, ∼0.5 kDa nucleotides) (48). In contrast, the segments of the VWF/fVIII coil evidently are the large ∼30–40 kDa intramonomeric domains. There is little precedent for a random coil with segments made up of folded, protein domains. One example is the mucin family of glycoproteins, which like VWF, consist of ∼0.5 MDa protomers with molecular weights extending to ∼20 MDa (49) and display MHKS relationships consistent with a random coil conformation (50). Like VWF, mucins are heavily O-glycosylated and contain a cysteine-rich domain homologous to the VWF C and D domains, indicating a common evolutionary origin of the flexibility of these proteins (51).

The unimodal molar mass distribution of unfractionated VWF/fVIII complexes (Fig. 5) is consistent with the mass distribution calculated by Ohmori *et al*. (5) from rotary shadowed EM images of VWF/fVIII multimers. They measured the contour length of extended multimers, modeled VWF as a cylinder using a particle density of 1.4 g/ml, and obtained a unimodal distribution, a median contour length of ∼400 nm, and a median molecular weight of ∼2 MDa. SEC showing an absorbance maximum with respect to elution volume (Fig. 2), which has been repeatedly observed during the purification of VWF/fVIII complexes (6, 7, 52, 53), also is consistent with a unimodal distribution. The results also are consistent with those of Lippok *et al*. (54) based on agarose gel electrophoresis, fluorescence correlation spectroscopy, and total internal reflection fluorescence microscopy in which the results were expressed as an exponential number-average distribution instead of as a molar mass distribution.

Multimers with molecular weight estimates extending to >20 MDa are evident by agarose gel electrophoresis of purified vWf/fVIII preparations and in normal plasma (3, 55). VWF multimers have been classified into molecular weight categories of low (0.5–2.5 MDa), intermediate (3–5 MDa), high (5–10 MDa), and ultralarge (>10 MDa), along with the proposal that only high-molecular-weight multimers support platelet adhesion and aggregation (56). Increased levels of ultralarge multimers resulting from deficiency of the metalloproteinase ADAMTS-13 are associated with the thrombotic disorder, thrombotic thrombocytopenic purpura (14, 15). Conversely, type 2A von Willebrand disease is a bleeding disorder associated with deficiency of high-molecular-weight multimers (3). The VWF/fVIII preparation in this study, with a median molecular weight of ∼2.2 MDa, is a therapeutic product with proven clinical efficacy in the management of von Willebrand disease (57). This suggests that either so-called low-molecular-weight multimers support platelet function or that most of the VWF mass in plasma is nonfunctional.

In shear flow, VWF multimers form “strings,” which have been proposed to be due to either extrusion from endothelial cells or self-association of multimers (13). Self-association of soluble VWF to immobilized VWF has been observed in shear flow (58, 59). Self-association manifests in the analytical ultracentrifuge as an increase in sedimentation coefficient with increased loading concentration. No evidence was found for self-association at concentrations of VWF/FVIII up to 1.1 mg/ml (Fig. 1*D*), which is over 100-fold greater than the plasma concentration of VWF/fVIII. Although it is possible that self-association is masked by strong solution nonideality, this seems unlikely in view of the molar mass distribution of unfractionated VWF complexes (Fig. 5), which indicates that the most common multimer is a ∼2.2 MDa 8-mer, considerably lower than the 250 monomers estimated for the longest strings (4).

The random coil behavior we observed in this study is not consistent with previous conclusions regarding the conformation of VWF under static conditions. Ohmori concluded that the presence of elongated VWF/fVIII multimers in EM images is inconsistent with a random coil conformation (5). The inability for an extended chain to adopt a random coil conformation implies a stiff, nearly rod-like structure. Examples of stiff chains include collagen (60) and low-molecular-weight DNA fragments (48), which are characterized by rod-like MHKS exponents (Table 3). Thus, it seems likely that the extended structures observed by EM are produced during their adsorption onto the imaging surface. Consistent with this, the same group reported that the most frequent imaged form was not extended, but rather a “loosely tangled coil”. Similarly, Slayter *et al*. imaged VWF/fVIII complexes by rotary-shadowed EM and found that the most frequently observed structures resembled a “loosely coiled ‘ball of yarn’” (16). Overall, the EM images of VWF seem consistent with a random coil adsorbed onto a two-dimensional surface. Singh *et al*. (40) analyzed the solution structure of unfractionated VWF/fVIII complexes by small-angle neutron scattering. They obtained a radius of gyration of 75 nm and modeled the structure as a prolate ellipsoid. This yielded values of 175 nm and 28 nm for the major and minor semiaxes and an axial ratio of 6.25. Although it is possible to calculate the axial ratio of a prolate ellipsoid given its radius of gyration, it does not follow that the particle resembles a prolate ellipsoid (37). The frictional ratio of a prolate ellipsoid is a function of its axial ratio and hydration. Within the range of hydration observed for proteins (0.2–0.5 g of water bound per gram protein), even relatively long, thin prolate ellipsoids produce frictional ratios only marginally greater than those found for globular proteins. For example, given an axial ratio of 6.25 reported by Singh *et al*., the frictional ratio is only 1.6 at maximal hydration (see Supporting information), which is significantly lower than the frictional ratio of ∼3 for VWF/fVIII (Table 1). Siedlecki *et al*. (41) obtained images of surface-bound VWF by atomic force microscopy and also modeled its structure as an ellipsoid. They obtained major and minor semiaxis values of 149 nm and 77 nm, which produce frictional ratios of only 1.2 at maximal hydration. Fu *et al*. (18) imaged dye-labeled recombinant VWF multimers by total internal reflection fluorescence microscopy and concluded that under static conditions, VWF is compact and globular. However, the resolution provided by this method does not allow differentiation between random coil and globular formations. Muller *et al*. (61) made AFM measurements on recombinant VWF dimers fixed between a poly-L-lysine-coated mica surface and a cantilever. In 6% of the force-extension traces, a 50–120 pN peak was observed that was interpreted as the result of disruption of an intermonomer force. However, in AFM imaging of the dimers under static conditions, 65% and 35% of the dimers appeared “flexible” and “closed,” respectively, which was construed as an equilibrium distribution. This indicates that intermonomer forces, if present, are not strong enough to overcome the transition to a flexible, random coil conformation produced by the thermal energy of the system. In a theoretical study, Sing and Alexander-Katz modeled VWF as a “dense globule” held together by intermonomer forces that unfolds during shear flow (19). As noted above, our results are inconsistent with a dense globular conformation.

A large body of evidence indicates that the interaction of VWF with the extravascular collagen, platelet GPIbα, and platelet αIIbβ3 is a function of shear stress in hemodynamic flow (4). VWF undergoes elongation during flow, which has been interpreted as being partly due to the disruption of interactions between monomers, resulting in unfolding of a collapsed multimer. However, random coils elongate in shear flow and can produce an abrupt coil-stretch transition (62). Thus, it is possible that shear stress acts solely to drive conformational changes within VWF domains that activate its binding functions. For example, the A1 domain elongates in shear flow into a conformation with high affinity for GPIbα (18). An autoinhibitory module has been identified that flanks and binds the A1 domain, deletion of which activates the A1 domain (63, 64), suggesting that shear flow produces dissociation of the autoinhibitory module. Additionally, shear flow has been reported to promote unfolding of the VWF A2 domain and drive self-association of VWF (59). In a random coil, all conformations have the same energy and are equally probable (20), suggesting that this plasticity allows for efficient activation in shear flow compared with the unfolding that would be required for a collapsed globule. Our results suggest that the random coil is the ground state conformation on which to build models of the activation of vWF/FVIII complex.

## Experimental procedures

Alphanate (antihemophilic factor/von Willebrand factor complex [human]) was purchased from Grifols USA. Amicon Ultra-4 and Ultra-15 centrifugal filters were from Merck Millipore. Sephacryl S-1000 was from GE Healthcare. Heparin-Sepharose was from Sigma Aldrich. Pooled citrated normal plasma (FACT) was from George King Biomedical. Protein extinction coefficients at 280 nm based on tryptophan, tyrosine, and cystine composition (65) and partial specific volumes based on amino acid composition (66) were calculated using SEDFIT version 16.36 (www.analyticalultracentrifugation.com). For VWF, the molecular weight, extinction coefficient, and partial specific volume were adjusted for a fractional carbohydrate composition of 0.187 using a polypeptide molecular weight of 225,388 Da (7, 8). A value of 0.622 ml/g was used for the glycan partial specific volume (67). A molecular weight for the VWF monomer of 277 kDa was calculated, yielding an extinction coefficient at 280 nm of 0.65 (mg/ml)^−1^ cm^−1^ and a partial specific volume of 0.706 ml/g. Lyophilized bovine serum albumin, fraction V RIA ELISA grade (BSA), was purchased from Calbiochem and dialyzed against 154 mM NaCl, 5.60 mM Na_2_HPO_4_, 1.1 mM KH_2_PO_4_, pH 7.40 (PBS) before use. A partial specific volume of 0.733 ml/g and extinction coefficient at 280 nm of 0.647 (mg/ml)^−1^ cm^−1^ were used for BSA. The solvent density and viscosity of 0.15 M NaCl, 0.02 M Hepes, 5 mM CaCl_2_, pH 7.4 (HBS/Ca Buffer), and PBS were estimated using SEDNTERP, version 1.10 (67).

### Purification of VWF/fVIII complexes

VWF/fVIII complex was purified from Alphanate, which is a commercial concentrate prepared from pooled human plasma by cryoprecipitation, fractional solubilization, and heparin-Sepharose affinity chromatography. Human albumin is added as a stabilizer. Albumin was removed by heparin-Sepharose chromatography as described in Supporting information (Fig. S1). The resulting product contained at least 90% VWF as judged by SDS-PAGE (Fig. S1). Heparin-Sepharose purified VWF/fVIII was concentrated to ∼1.5 mg/ml using an Amicon Ultra-4 100 kDa membrane. For SV AUC, heparin-Sepharose purified VWF/fVIII was dialyzed overnight against HBS/Ca Buffer and diluted in dialysate to the concentrations indicated in text.

### Size-exclusion chromatography (SEC) of VWF/fVIII complexes

Heparin-Sepharose purified VWF/fVIII (17 ml, 1.5 mg/ml) was applied to a 2.5 × 120 cm Sephacryl S1000 SEC column equilibrated in 0.08 M NaCl, 0.02 M MES, 5 mM CaCl_2_, 1 M glycerol, pH 6.2 at room temperature. Fractions were collected at a flow rate of 35 ml/min. Pooled fractions were collected and designated Samples A, B, C, D, and E, respectively. The samples were buffer-exchanged by exhaustive ultrafiltration into HBS/Ca Buffer and concentrated to 1.5–1.8 mg/ml using Amicon Ultra-4 100 kDa centrifugal filters and frozen at –80 °C. For SV AUC, the samples were thawed in a 37 °C water bath for 15 min, centrifuged at 2000*g* for 30 s, and diluted with HBS/Ca Buffer. For DLS, samples were thawed in a 37 °C water bath for 15 min, centrifuged at 18,000*g* for 30 min, and the supernatant was removed for analysis.

### Dynamic light scattering (DLS)

DLS measurements were made using a Zetasizer Nano S system (Malvern Pananalytical) at a scattering angle of 173 degrees. Measurements of the normalized electric field correlation function, $g_{1}\left(\tau \right)$, as a function of decay time, *τ*, were made on 0.02 ml samples at 20 °C in a 3 mm ZEN2112 quartz cuvette in automatic attenuation mode. Eight measurements were made on each sample.

For a polydisperse system, $g_{1}\left(\tau \right)$ is characterized by a distribution of exponential decay rates, G(Γ), given by

(1) $$g_{1}\left(\tau \right)=\overset{\infty }{\underset{0}{\int }}\text{G}\left(\text{Γ}\right)\exp\left(-\text{Γ}\tau \right)d\text{Γ}$$

(68). The decay rate is related to the translational diffusion coefficient, *D*, by

(2) $$\text{Γ}=Dq^{2}$$

where *q* is the magnitude of the scattering vector

(3) q=4πλsin(θ2)

*θ* is the scattering angle, λ=λon, $\lambda_{o}$ is the vacuum wavelength of incident light, and *n* is the solvent index of refraction.

G(Γ) distributions derived from SEC samples initially were estimated by fitting $g_{1}\left(\tau \right)$ *versus τ* using the continuous I(D)—distribution model in SEDFIT as described (69) and were monomodal. The first, second, and third moments of the distribution, representing the mean ($\mu_{1}=\bar{\text{Γ}}$), variance (*μ*_2_), and skewness (*μ*_3_) of G(Γ), were estimated by the method of cumulants (68, 70), which is applicable to monomodal, polydisperse distributions, using the cumulant analysis model in SEDFIT. $\bar{\text{Γ}}$ is the z-average diffusion coefficient, *D*_*z*_ (70),

(4) $$D_{z}=\underset{k}{\sum }c_{k}m_{k}D_{k}/\underset{k}{\sum }c_{k}m_{k}$$

where *c*_*k*_, *m*_*k*_, and *D*_*k*_ are the total cell concentration, mass, and diffusion coefficient of species *k*.

Diffusion coefficients were adjusted to the standard condition of 20 °C in solvent water using

(5) $$\frac{D}{D_{20,w}}=\frac{T}{T_{20}}\frac{\eta_{20,w}}{\eta }$$

where *T* and *T*_20_ are the absolute experimental temperature and temperature at 20 °C, and *η* and *η*_20,*w*_ are the corresponding solvent viscosities (20). The z-average diffusion coefficient under standard conditions is then ${\left(D_{z}\right)}_{20,w}$.

Confidence limits for ${\left(D_{z}\right)}_{20,w}$ based on the eight measurements were calculated using Student's *t* distribution. The polydispersity index was calculated using (35)

(6) $$PDI=\frac{\mu_{2}}{{\bar{\text{Γ}}}^{2}}$$

### Hydrodynamic radii and frictional ratios

The hydrodynamic (Stokes) radius of a particle, *R*_*h*_ is defined as the radius of an anhydrous spherical particle that has same diffusion coefficient that of the subject particle. *R*_*h*_ was calculated using the Stokes–Einstein equation (68)

(7) $$R_{h}=\frac{k_{B}T}{6\pi \eta D}$$

where *k*_*B*_ is the Boltzmann constant. Frictional coefficients were calculated from the measured diffusion coefficient using the Einstein diffusion equation (68, 71)

(8) $$D=\frac{k_{B}T}{f}$$

Frictional ratios, *f*/*f*_*o*_, were calculated using (72)

(9) $$f_{o}=6\pi \eta {\left(\frac{3M\bar{\nu }}{4\pi N_{A}}\right)}^{\frac{1}{3}}$$

where *f*_*o*_is the frictional coefficient of the equivalent sphere having the same anhydrous molecular weight and partial specific volume, $\bar{\nu }$, of the particle, *M* is the molar mass estimated using the Svedberg equation (Equation 14), and *N*_*A*_ is Avogadro's number.

### Sedimentation velocity analytical ultracentrifugation (SV AUC)

SV AUC was conducted at a nominal temperature of 20 °C in a Beckman Coulter XLI analytical ultracentrifuge using standard procedures (73). Samples (0.4 ml) were loaded into 12 mm pathlength Epon double sector cells equipped with sapphire windows with matched buffer in the reference sector. A small correction for temperature was made by direct measurement of the rotor temperature as described (74). Data were corrected for scan time errors using REDATE version 1.01 (75). Absorbance scans at 280 nm and interference scans were initiated after reaching the target rotor speeds.

Data were analyzed using the continuous *c*(*s*) distribution model (24, 25, 26) in SEDFIT by fitting to the integral equation (see Supporting information):

(10) $$S\left(r,t\right)=\overset{s_{max}}{\underset{s_{min}}{\int }}c\left(s\right)\chi_{1}\left(s,f/f_{o},r,t\right)ds+b\left(r\right)+\epsilon_{systematic}+\epsilon_{random}$$

where *S*(*r*,*t*) is the absorbance or interference signal calculated from the model, *r* is the radius from the center of rotation, *t* is time of centrifugation, *s* is the sedimentation coefficient, *f*/*f*_*o*_ is the frictional ratio, *b*(*r*) is the baseline, $\epsilon_{systematic}$ is the sum of the time-invariant noise, $\epsilon_{TI}$ (for absorbance and interference data), and radial-invariant noise, $\epsilon_{RI}$ (for interference data), and $\epsilon_{random}$ is random noise. $\chi_{1}\left(s,f/f_{o},r,t\right)$ is the signal produced by a species with sedimentation coefficient s and diffusion coefficient *D* loaded at unit signal strength, which serves to normalize the signals produced by the various species (76). Data also were analyzed using the continuous *c*(*M*) distribution model (24) in SEDFIT by fitting the integral

(11) $$S\left(r,t\right)=\overset{M_{max}}{\underset{M_{min}}{\int }}c\left(M\right)\chi_{1}\left(M,f/f_{o},r,t\right)dM+b\left(r\right)+\epsilon_{systematic}+\epsilon_{random}$$

*c*(*s*) and *c*(*M*) integrals were discretized into 100 intervals over a range of 0–80 S and 30 intervals over a range of 0 to 6 × 10^6^ g/mol, respectively. *f*/*f*_*o*_ was a fitted parameter in the *c*(*s*) integral and held fixed at 3.0 in the *c*(*M*) integral. The other fitted parameters were *c*(*s*), *c*(*M*), $\epsilon_{TI}$, $\epsilon_{RI}$, and the meniscus position, which is a boundary condition in the integral equations. Fitting was done using sequential simplex and Marquardt–Levenberg algorithms and maximum entropy regularization with a confidence interval of 0.68. SV graphs were plotted using GUSSI version 1.2.1 (77).

The weight-average sedimentation coefficient, *s*_*w*_, is given by

(12) $$s_{w}=\underset{k}{\sum }c_{k}s_{k}/\underset{k}{\sum }c_{k}$$

where *c*_*k*_ and *s*_*k*_ are the total cell concentration and sedimentation coefficient of species *k* (27, 78). *s*_*w*_ values were estimated by integrating c(s) distributions from 8 to 80 S. Sedimentation coefficients were adjusted to the standard condition of 20 °C in solvent water using

(13) $$\frac{s_{w}}{{\left(s_{w}\right)}_{20,w}}=\frac{\eta_{20,w}}{\eta }\frac{\left(1-\bar{\nu }\rho \right)}{\left(1-\bar{\nu }\rho_{20,w}\right)}$$

where the partial specific volume is assumed invariant with respect to solvent conditions, and *ρ* and *ρ*_20,*w*_ are the experimental density and density of water at 20 °C (20). Confidence limits for *s*_*w*_ values were calculated using Monte Carlo simulation as described in Supporting information.

### Molar mass

Molar masses were estimated using the Svedberg equation

(14) $$M=\frac{sRT}{D\left(1-\bar{\nu }\rho \right)}$$

where *R* is the gas constant (79). The value used for *s* was the value at infinite dilution under standard conditions, ${\left(s_{w}^{0}\right)}_{20,w}$, which was estimated by regression analysis of a plot of ${\left(s_{w}\right)}_{20,w}$ *versus* concentration extrapolated to zero concentration (Fig. 1*D*). The value used for *D* was the value at infinite dilution under standard conditions, ${\left(D_{z}^{0}\right)}_{20,w}$, was assumed to be equal to the experimental value at 0.5 mg/ml, since no concentration dependence in *D*_*z*_was observed from 0.5 to 1.0 mg/ml.

### Mark–Houwink–Kuhn–Sakurada (MHKS) parameters

MHKS exponents for sedimentation

(15) $$s=k^{\prime }M^{a_{s}}$$

and diffusion

(16) $$D=k^{\prime \prime }M^{a_{D}}$$

were estimated from the regression of log_10_ ${\left(s_{w}^{0}\right)}_{20,w}$ and log_10_ ${\left(D_{z}^{0}\right)}_{20,w}$ on log_10_ *M*.

### Factor VIII assay

FVIII activity was measured by one-stage coagulation assay using a Diagnostica Stago Start viscosity-based coagulation analyzer and referenced to pooled citrated normal human plasma as described (80).

## Data availability

Raw data files are available upon request from Pete Lollar (jlollar@emory.edu). All remaining data are contained within the article.

## Supporting information

This article contains supporting information.

## Conflict of interest

The authors declare that they have no conflicts of interest with the contents of this article.
