# Supplementary material for: Conformation of the von Willebrand factor/factor VIII complex in quasi-static flow
Source: J Biol Chem. 2021 Feb 16;296:100420. doi: 10.1016/j.jbc.2021.100420 (PMC8005835; doi:10.1016/j.jbc.2021.100420)
Supplement: Supporting information [file mmc1.pdf]

Conformation of the von Willebrand factor/factor VIII complex in quasi-static flow

Supporting Information

Ernest T. Parker[1], Pete Lollar[1]*

[1]Aflac Cancer and Blood Disorders Center, Children's Healthcare of Atlanta; Department of Pediatrics, Emory University, Atlanta, GA.

*Corresponding author: Pete Lollar
E-mail: jlollar@emory.edu

Purification of VWF/fVIII complexes by heparin-Sepharose chromatography

Two vials of Alphanate lot B1NEA00092 were reconstituted with 10 mL sterile water for injection per vial, diluted with an equal volume of 0.125 M NaCl, 20 mM Hepes, pH 7.4 and loaded onto a 2.5 x 12.5 cm heparin-Sepharose column equilibrated in the same buffer at 2 mL/min using a Bio-Rad Low Pressure Chromatography system (Fig. S1). The column was washed to undetectable A280 in the same buffer. Albumin was eluted with a 0.125 M to 0.15 M NaCl gradient in 20 mM Hepes, pH 7.4. VWF-fVIII was eluted with a 0.15 M to 0.6 M NaCl gradient in 20 mM Hepes, pH 7.4. Fractions 26-38 were pooled, $CaCl_2$ was added to a final concentration of 5 mM and the preparation was concentrated to 45 ml using an Amicon Ultra-15 100 kDa filter.

The continuous c(s) distribution model

The continuous c(s) distribution model in SEDFIT assumes a continuously polydisperse system of non-interacting species in an ideal solution (1-3). Each species k has sedimentation and translational diffusion coefficients s_k and D_k, and a time- and radial-dependent concentration $c_k(r,t)$ in a sector-shaped centrifuge cell described by the Lamm equation ((4), Eq 11-7, p. 598) :

$$\frac{\partial c_k(r,t)}{\partial t} = \frac{1}{r}\frac{\partial}{\partial r}\left[rD_k\frac{\partial c_k(r,t)}{\partial r} - s_k\omega^2 r^2 c_k(r,t)\right] \quad (1)$$

where t is time of centrifugation, r is the radius from the center of rotation, and ω is the angular velocity of the rotor. Each species produces a dimensionless absorbance and interference signal given by

$$\chi_k = \varepsilon_k l c_k \quad (2)$$

where ε_k is the extinction coefficient or interference fringe coefficient and l is the optical pathlength. Because the Lamm equation is a mass transport equation, the units of ε_k are mass-based (e.g., $(\text{mL/mg})^{-1}\text{cm}^{-1}$). For a homologous series of polymers such as VWF multimers, absorbance and interference ε_k values are assumed constant and equal to that of the monomer. Substituting, ε_k and l cancel, and

$$\frac{\partial\, \chi_k(r,t)}{\partial t} = \frac{1}{r}\frac{\partial}{\partial t}\left[rD_k\frac{\partial\, \chi_k(r,t)}{\partial r} - s_k\omega^2 r^2\, \chi_k(r,t)\right] \quad (3)$$

The summation of Lamm equation solutions for all species produces a signal given by (5):

$$S'(r,t) = \int_{D_{min}}^{D_{max}}\int_{s_{min}}^{s_{max}} c(s,D)\chi_1(s,D,r,t)ds\, dD \quad (4)$$

where $c(s,D)$ is the sedimentation-diffusion coefficient distribution and $\chi_1(s,D,r,t)$ is the normalized signal produced by a species with sedimentation coefficient s and diffusion coefficient D loaded at unit signal strength (6).

The key assumption in the continuous $c(s)$ distribution model is that all species have a common frictional ratio, f/f_o, where f is the frictional coefficient and f_o is the frictional coefficient of an equivalent sphere having the same anhydrous molar mass and specific volume as the sedimenting particle. The assumption is motivated by the fact that for systems of macromolecules with similar shape or conformation, f/f_o does not very widely and also because suboptimal values of f/f_o have little effect on the location of $c(s)$ peaks (2). The model also assumes that the partial specific volume is known and constant for all species. The partial specific volume of the VWF multimer is expected to be constant equal to that of the monomer, which can be estimated from the amino acid and carbohydrate composition. The diffusion coefficient is eliminated in favor of f/f_o using (2)

$$D = \frac{\sqrt{2}}{18\pi}k_B T\, s^{-1/2}[\,\eta(f/f_o)\,]^{-3/2}\left(\frac{\bar{v}}{1-\bar{v}\rho}\right)^{-1/2} \quad (5)$$

where $\bar{\nu}$ is the partial specific volume, k_B is the Boltzmann constant, T is temperature, η is solvent viscosity and ρ is solvent density. This equation can be derived from standard textbook equations as follows. The frictional coefficient is given by the Einstein diffusion equation ((7), Eq 10-66, p. 583)

$$D = \frac{k_B T}{f} \tag{6}$$

which can be re-written

$$D = \frac{k_B T}{(f/f_o) f_o} \tag{7}$$

Using ((7), p. 585)

$$f_o = 6\pi\eta \left(\frac{3M\bar{\nu}}{4\pi N_A}\right)^{\frac{1}{3}} \tag{8}$$

where N_A is Avogadro's number and $\bar{\nu}$ is considered equal to the specific volume of the particle, substitution yields

$$D = \frac{k_B T}{6\pi\eta (f/f_o) \left(\frac{3}{4\pi}\frac{M}{N_a}\bar{\nu}\right)^{1/3}} \tag{9}$$

The Svedberg equation is ((4), Eq 11-33, p. 607)

$$M = \frac{sRT}{D(1-\bar{\nu}\rho)} \tag{10}$$

where R is the gas constant. Substituting for M in Equation 9 and using $R = N_A k_B$ yields

$$D = \frac{k_B T}{(f/f_o) 6\pi\eta \left(\frac{3 k_B T s \bar{\nu}}{4\pi D(1-\bar{\nu}\rho)}\right)^{\frac{1}{3}}} \tag{11}$$

Solving for D yields the desired relationship.

Thus, the distribution of sedimentation coefficients, $c(s)$, is given by (8)

$$S'(r,t) = \int_{s_{min}}^{s_{max}} c(s)\chi_1(s, f/f_o, r, t) ds \tag{12}$$

The calculated signal, $S(r,t)$, also includes a baseline, $b(r)$, and noise

$$S(r,t) = \int_{s_{min}}^{s_{max}} c(s)\chi_1(s, f/f_o, r, t) ds + b(r) + \epsilon_{systematic} + \epsilon_{random} \tag{13}$$

where $\epsilon_{systematic}$ is the sum of the time-invariant noise, ϵ_{TI} (for absorbance and interference data), and radial-invariant noise, ϵ_{RI} (for interference data), and ϵ_{random} is random noise (9). SEDFIT divides the integral in Eq 13

into a user-defined set of discrete intervals of s and solves the Lamm equation numerically as a function of the fitted parameters $c(s)$, f/f_o, and the meniscus position, which is a boundary condition. It minimizes the difference between the experimental signal, $a(r,t)$, and calculated values of the discretized $S(r,t)$ according to

$$\min_{\{p\}} \sum_{r,t} \big(a(r,t) - S(r,t)\big)^2 \tag{14}$$

where the fitted parameters $\{p\}$ also include ϵ_{TI}, ϵ_{RI}, $b(r)$ (3). The integral in Equation 13 is a Fredholm integral that is inverted to produce the $c(s)$ distribution. Experimental noise leads to overfitting the data and spurious spikes in the $c(s)$ distribution. This is problem is addressed by maximum entropy or Tikhonov–Phillips regularization, which seeks to produce the most parsimonious distribution function that is consistent with the data (1, 2).

The 95% confidence limits for s_w values were calculated by Monte Carlo simulation as described ((3), p. 200). First, the 95% confidence limits for the meniscus position were calculated using the Calculate Variance Ratio with F-Statistics function in SEDFIT. Then 95% confidence limits at each meniscus limit were calculated using the Monte-Carlo for Integrated Weight-Average s-Values function in SEDFIT using 1000 simulations at each limit. The outer range of the four limits was used to produce the confidence limits.

Frictional ratios of a prolate ellipsoid

The frictional ratio of a prolate ellipsoid is given by ((7), Eq 10-70, p. 585)

$$\frac{f}{f_o} = \left(\frac{(\bar{v} - \delta_1 v_w)}{\bar{v}}\right)^{1/3} F \tag{15}$$

where $\bar{v}$ is the partial specific volume of the ellipsoid, v_w is the specific volume of water, δ_1 is the hydration of the particle in grams water bound per gram of particle and F is the Perrin factor,

$$F = \frac{\left(1 - {b^2}/{a^2}\right)^{1/2}}{\left({b}/{a}\right)^{2/3} ln\left(\frac{1 + \left(1 - {b^2}/{a^2}\right)^{1/2}}{b/a}\right)} \tag{16}$$

where a and b are the lengths of the major and minor semi-axes ((7), Eq 10-19a, p. 561). Singh et al. reported values of 175 nm and 28 nm for a and b (10), which produces a Perrin factor of 1.33. Using $\bar{v}$ equal to 0.706 mL/g for VWF/fVIII and δ_1 ranging from 0.2 to 0.5 g/g, produces ${f}/{f_o}$ values ranging from 1.4 to 1.6.

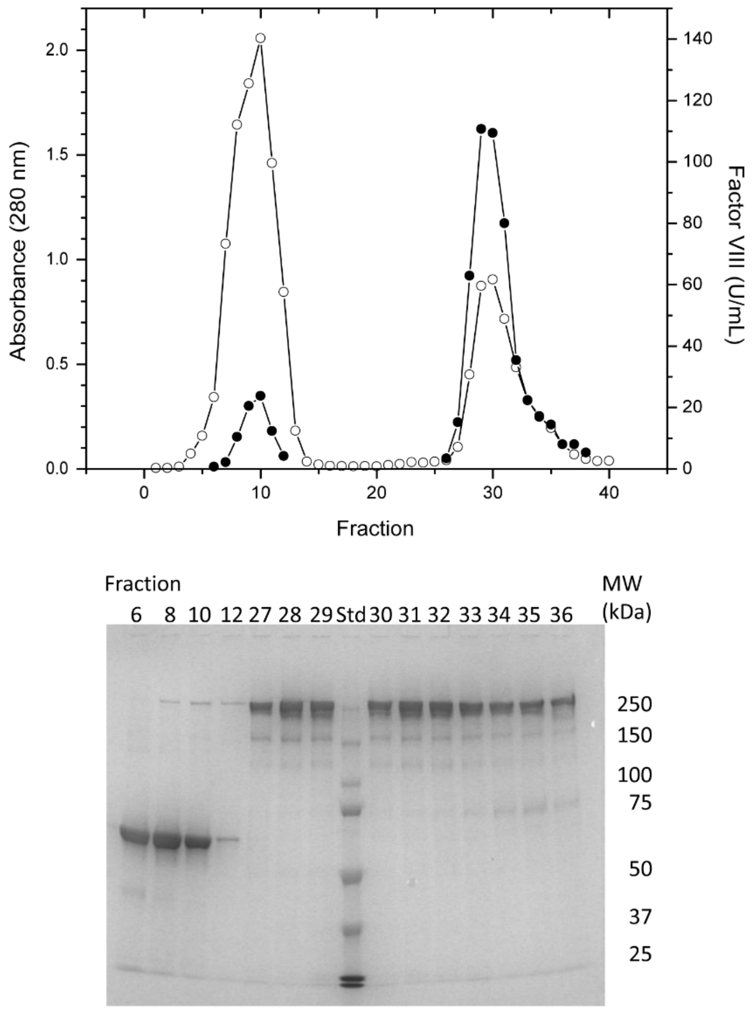

Fig. S1. Isolation of VWF/fVIII from Alphanate by heparin-Sepharose chromatography. Fractions 1 – 4, load and fall-through; fractions 5 – 15, 0.125 M to 0.15 M NaCl gradient in 0.02 M Hepes, pH 7.4; fractions 15 – 40, 0.15 M to 0.60 M NaCl gradient in 0.02 M Hepes, pH 7.4. Open circles, A280; closed circles, fVIII activity. Fraction volumes, 10 mL. Lower panel, 7.5% SDS-PAGE under reducing conditions, ~3 µg/lane.

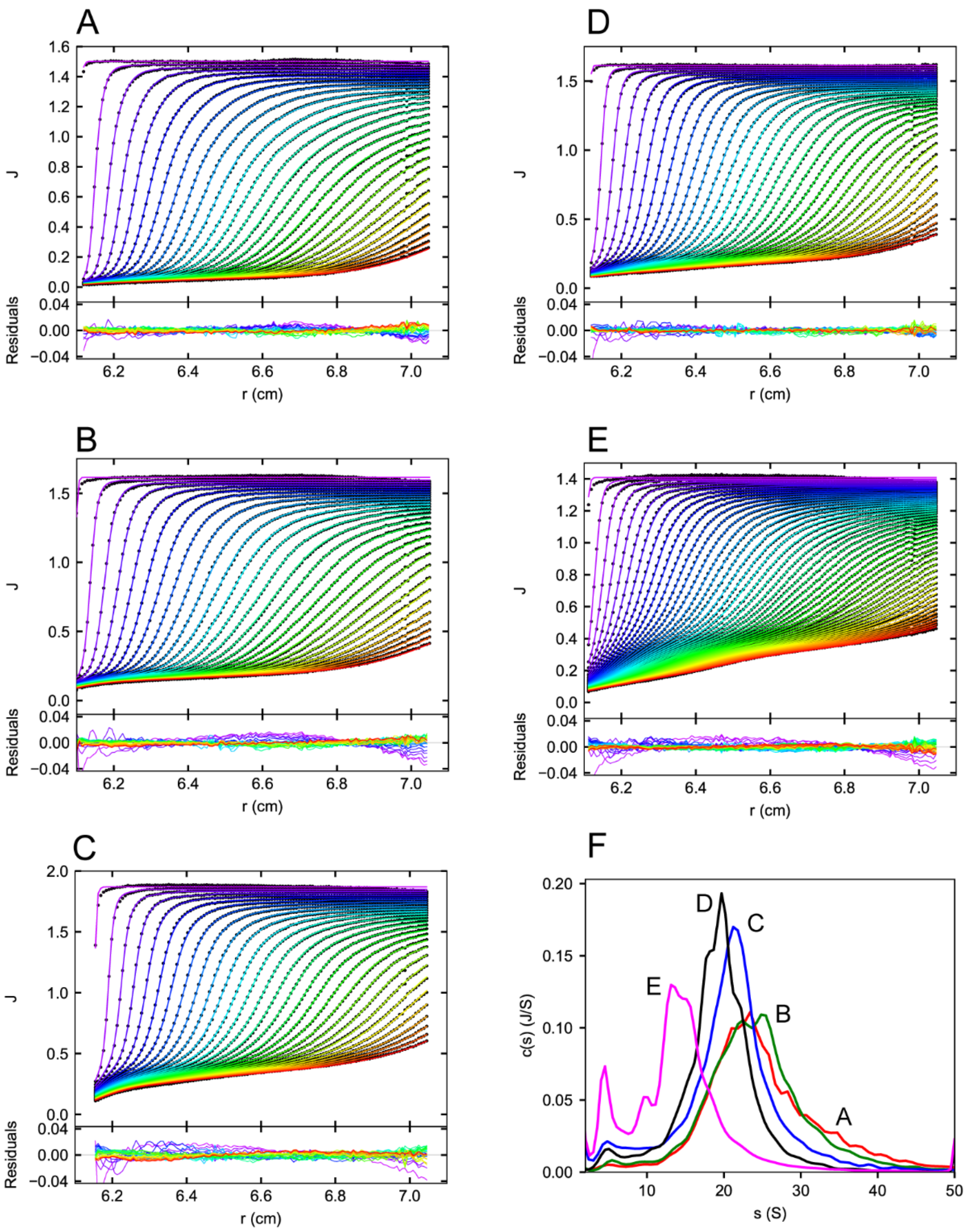

Fig. S2. SV AUC of SEC-fractionated VWF/fVIII complexes - interference optics. Samples A, B, C, D and E in HBS/Ca Buffer were centrifuged at 45,400*g*. *Panels A-E*: Interference scans; J, fringe increment. Curves represent fits to the continuous c(s) distribution model. Only every tenth data point is shown for clarity. The root-mean-square-deviations of the fits ranged from 0.0031 to 0.0056. *Panel F*: c(s) distributions derived from the fitted data.

Table S1 Hydrodynamic properties of globular proteins

Protein	$MW\ (Da)$	$s_{20,w}$ (S)	$\bar{v}$ (mL/g)	f/f_0	D (cm^2/s)	$\frac{s\bar{v}^{1/3}}{(1-\bar{v}\rho)}$ (S cm/g^3)	R_h (nm)
Lipase	6.67E+03	1.14	0.714	1.20	1.45E-06	3.55	1.5
Insulin dimer	1.15E+04	1.60	0.744	1.08	1.32E-06	5.63	1.6
Cytochrome c	1.24E+04	1.80	0.707	1.18	1.20E-06	5.45	1.8
RNAse A	1.37E+04	1.78	0.696	1.33	1.04E-06	5.17	2.1
Lysozyme	1.43E+04	1.91	0.702	1.24	1.09E-06	5.67	2.0
α-lactalbumin	1.42E+04	1.92	0.704	1.22	1.11E-06	5.75	1.9
Myoglobin	1.78E+04	1.98	0.741	1.19	1.04E-06	6.88	2.1
Papain	2.34E+04	2.42	0.723	1.25	9.08E-07	7.80	2.4
α-chymotrypsin	2.50E+04	2.40	0.736	1.25	8.82E-07	8.17	2.4
Chymotrypsinogen A	2.58E+04	2.58	0.736	1.19	9.20E-07	8.78	2.3
Elastase	2.59E+04	2.60	0.730	1.21	9.02E-07	8.63	2.4
Subtilisin BPN	2.55E+04	2.77	0.731	1.12	9.78E-07	9.23	2.2
Carbonic anhydrase	3.00E+04	3.00	0.735	1.14	9.15E-07	10.17	2.3
**vWf A1 1272-1458**	**3.15E+04**	**2.50**	**0.723**	**1.52**	**---**	**8.06**	**---**
Riboflavin-binding protein	3.25E+04	2.76	0.720	1.39	7.36E-07	8.79	2.9
Carboxypeptidase	3.45E+04	3.20	0.725	1.22	8.19E-07	10.40	2.6
Pepsin	3.42E+04	2.88	0.725	1.35	7.44E-07	9.36	2.9
β-lactoglobulin A dimer	3.67E+04	2.87	0.751	1.27	7.61E-07	10.42	2.8
β-lactoglobulin A	3.50E+04	3.08	0.750	1.15	8.53E-07	11.13	2.5
Kinesin motor domain	4.14E+04	3.25	0.733	1.31	7.13E-07	10.92	3.0
Albumin ovum	4.40E+04	3.60	0.740	1.20	7.63E-07	12.46	2.8
BSA	6.63E+04	4.50	0.735	1.29	6.21E-07	15.25	3.5
Hemoglobin (human)	6.45E+04	4.60	0.749	1.16	6.89E-07	16.55	3.1
Anthrax protective antigen	8.50E+04	5.01	0.762	1.21	6.00E-07	19.12	3.6
LDH (dogfish)	1.38E+05	7.54	0.741	1.22	5.10E-07	26.21	4.2
β-lactoglobulin A octamer	1.47E+05	7.38	0.751	1.24	4.89E-07	26.79	4.4
GPD apoprotein	1.43E+05	7.60	0.737	1.26	4.90E-07	25.97	4.4
Aldolase	1.56E+05	7.40	0.742	1.34	4.46E-07	25.83	4.8
Malate synthetase	1.70E+05	8.25	0.735	1.31	4.44E-07	27.96	4.8
Catalase	2.48E+05	11.3	0.730	1.26	4.09E-07	37.50	5.2
Glutamate dehydrogenase	3.12E+05	11.4	0.749	1.34	3.53E-07	41.03	6.1
Apoferritin	4.67E+05	17.6	0.750	1.13	3.65E-07	63.62	5.9
Apoferritin (horse spleen)	5.02E+05	18.3	0.728	1.26	3.25E-07	60.23	6.6
Urease	4.83E+05	18.6	0.742	1.13	3.62E-07	64.93	5.9
Glutamate dehydrogenase	1.02E+06	26.6	0.730	1.37	2.35E-07	88.28	9.1
Hemoglobin (snail)	3.50E+06	58.9	0.747	1.31	1.61E-07	210.12	13.3
Hemocyanin	8.95E+06	105.8	0.728	1.48	1.05E-07	348.24	20.3

From (11), Table D2.3.
Bold: from (12).

1. Schuck P. Size-distribution analysis of macromolecules by sedimentation velocity ultracentrifugation and Lamm equation modeling. Biophysical Journal. 2000;78(3):1606-19.
2. Dam J, Schuck P. Calculating sedimentation coefficient distributions by direct modeling of sedimentation velocity concentration profiles. Methods in Enzymology. 2004;384:185-212.
3. Schuck P. Sedimentation Velocity Analytical Ultracentrifugation. Boca Raton: CRC Press; 2017.
4. Cantor CR, Schimmel PR. Ultracentrifugation. Biophysical Chemistry Part II Techniques for the Study of Biological Structure and Function. San Francisco: W.H. Freeman and Co.; 1980. p. 591-642.
5. Schuck P. Analytical ultracentrifugation as a tool for studying protein interactions. Biophysical Review. 2013;5(2):159-71.
6. Dam J, Velikovsky CA, Mariuzza RA, Urbanke C, Schuck P. Sedimentation velocity analysis of heterogeneous protein-protein interactions: Lamm equation modeling and sedimentation coefficient distributions c(s). Biophysical Journal. 2005;89(1):619-34.
7. Cantor CR, Schimmel PR. Size and shape of macromolecules. Biophysical Chemistry Part II Techniques for the Study of Biological Structure and Function. San Francisco: W.H. Freeman and Co.; 1980. p. 539-90.
8. Dam J, Schuck P. Sedimentation velocity analysis of heterogeneous protein-protein interactions: sedimentation coefficient distributions c(s) and asymptotic boundary profiles from Gilbert-Jenkins theory. Biophysical Journal. 2005;89(1):651-66.
9. Schuck P, Demeler B. Direct sedimentation analysis of interference optical data in analytical ultracentrifugation. Biophysical Journal. 1999;76(4):2288-96.
10. Singh I, Shankaran H, Beauharnois ME, Xiao ZH, Alexandridis P, Neelamegham S. Solution structure of human von Willebrand factor studied using small angle neutron scattering. Journal of Biological Chemistry. 2006;281(50):38266-75.
11. Zaccai NR, Serdyuk IN, Zaccai J. Hydrodynamics. Methods in Molecular Biophysics. 2nd ed. Cambridge: Cambridge University Press; 2017. p. 159-250.
12. Deng W, Wang Y, Druzak SA, Healey JF, Syed AK, Lollar P, et al. A discontinuous autoinhibitory module masks the A1 domain of von Willebrand factor. Journal of Thrombosis and Haemostasis. 2017;15(9):1867-77.
